# Oxidative Stability of Fish Oil-Loaded Nanocapsules Produced by Electrospraying Using Kafirin or Zein Proteins as Wall Materials

**DOI:** 10.3390/antiox13091145

**Published:** 2024-09-23

**Authors:** Nor E. Rahmani-Manglano, Elnaz Z. Fallahasghari, Ana C. Mendes, Mogens L. Andersen, Emilia M. Guadix, Ioannis S. Chronakis, Pedro J. García-Moreno

**Affiliations:** 1 Department of Chemical Engineering, University of Granada, 18071 Granada, Spain; norelenarm@ugr.es (N.E.R.-M.); eguadix@ugr.es (E.M.G.); 2 DTU-Food, Research Group for Food Production Engineering, Laboratory of Nano-BioScience, Technical University of Denmark, Henrik Dams Allé, B202, 2800 Kongens Lyngby, Denmark; zeyfal@food.dtu.dk; 3 Department of Food Science, University of Copenhagen, 1958 Copenhagen, Denmark; mola@food.ku.dk

**Keywords:** lipid oxidation, omega-3 polyunsaturated fatty acids, encapsulation, prolamins, kafirin, zein, electron paramagnetic resonance, differential scanning calorimetry

## Abstract

The encapsulation of fish oil by monoaxial electrospraying using kafirin or zein proteins as hydrophobic wall materials was investigated. Kafirin resulted in spherical fish oil-loaded nanocapsules (>50% of capsules below 1 µm), whereas zein led to fish oil-loaded nanocapsules with non-spherical morphology (>80% of capsules below 1 µm). Both hydrophobic encapsulating materials interacted with fish oil, successfully entrapping the oil within the protein matrix as indicated by Fourier-transform infrared spectroscopy (FTIR) and Raman spectroscopy results. FTIR also suggested hydrogen bonding between fish oil and the proteins. Trapped radicals in the encapsulation matrix that were detected by electron paramagnetic resonance (EPR), indicated oxidation during electrospraying and storage. Results from isothermal (140 °C) differential scanning calorimetry (DSC) denoted that the encapsulation of fish oil by electrospraying using both kafirin or zein as wall materials protected fish oil from oxidation. In particular, the zein-based nanocapsules were 3.3 times more oxidatively stable than the kafirin-based nanocapsules, which correlates with the higher oil encapsulation efficiency found for zein-based capsules. Thus, this study shows that kafirin might be considered a hydrophobic wall material for the encapsulation of fish oil by electrospraying, although it prevented lipid oxidation to a lower extent when compared to zein.

## 1. Introduction

The populations of most countries in the world present a low or considerably low omega-3 index (Schuchardt et al., 2024), which is associated with an increasing risk of chronic diseases (e.g., cardiovascular and inflammatory diseases) [1]. Therefore, the food industry aims to develop omega-3-enriched food, which could raise the omega-3 level in those populations with low consumption of natural omega-3-rich food products (e.g., fatty fish, krill, some microalgae species) [2]. Fish oil, which is rich in omega-3 polyunsaturated fatty acids such as eicosapentaenoic (C20:5n-3, EPA) and docosahexaenoic (C22:6n-3, DHA) acids, is the most common ingredient used to produce omega-3-fortified food products [3]. Nevertheless, EPA and DHA are highly prone to oxidation, leading to rancidity and altering the nutritional and organoleptic properties of foods [4].

The encapsulation of fish oil, consisting of the entrapment of the oil within biopolymers wall material(s), is widely carried out by the food industry to (i) enhance the incorporation of lipophilic ingredients into aqueous-based food matrices, (ii) increase the oxidative stability of the resulting fortified product, and (iii) improve the target delivery of the bioactive in the small intestine [5,6]. Electrospraying, which allows drying at room temperature, which can mitigate lipid oxidation, has been reported as a relevant encapsulation technique used to produce omega-3 nano-microencapsulates [7]. In electrospraying, a high-voltage electrostatic field is applied between an injector and a collector charging the surface of the feed solution, which leads to the formation of a Taylor cone, the ejection of a jet, jet destabilization into charged droplets due to the low viscoelasticity of the feed solution, and solvent evaporation in the way to the collector, where dried particles are obtained [8,9]. In particular, the formation of a polymer jet is mainly governed by Coulombic, electric field, viscoelastic, and surface tension forces and other forces, such as air drag and gravitational forces. Depending on the relative magnitude of viscoelastic and surface tension, the jet can be deposited as electrospun fibres or broken into liquid droplets forming electrosprayed particles [10]. Nevertheless, electrosprayed capsules loaded with omega-3 have been mainly produced with hydrophilic polysaccharides (maltodextrin, glucose syrup, dextran, Arabic gum) and/or aqueous-soluble proteins (whey proteins, soy proteins, gelatin) as main encapsulating agents [7,11]. The use of an aqueous-soluble encapsulating agent results in potential capsule disintegration when incorporating these encapsulates into aqueous-based food matrices, which does not enhance the oxidative stability of the encapsulated fish oil [12].

Protein-based materials provide various advantages as encapsulating agents when compared with carbohydrates, such as a high binding capacity, the availability of a large surface area for entrapment, and an ability to interact with molecules of different polarities based on pH with electrostatic interaction or hydrophobic bonds [13]. In particular, zein, a prolamin obtained from maize displays significant hydrophobic properties and has been widely investigated as wall material for the encapsulation of fish oil by electrospraying [14,15,16]. In fact, in our previous study [16], we confirmed that fish oil-loaded electrosprayed capsules produced with zein as the encapsulating agent remained intact after their incorporation into an aqueous-based food matrix such as mayonnaise, improving the oxidative stability of the omega-3-fortified product. However, zein has been reported to present several maize impurities, which might lead to undesired colour, odour, or flavour, limiting its use as an encapsulating material [8]. Therefore, further research on the use of alternative prolamin proteins to encapsulate fish oil is required.

Sorghum, which is a drought-tolerant crop that properly adapts to hot and arid regions, is gaining interest in Europe not only for bio-alcohol production or animal feed but also for food applications [17,18]. Indeed, protein content in sorghum grains ranges between 6 and 18 wt.%, serving as a source of natural and sustainable plant protein-based ingredients [18]. Interestingly, the most abundant protein in sorghum grain is kafirin (making up 70–90% of the total), a food storage prolamin with α-, β-, and γ-subunits with similar molecular weight and structure when compared to zein [19,20]. Moreover, kafirin presents higher cysteine content than zein, resulting in higher hydrophobicity due to the higher formation of disulfide crosslinks [21]. In addition, and contrarily to other grain prolamins (e.g., wheat, rye or barley), kafirin is non-allergic and thus safe for consumers with celiac disease [22].

Kafirin particles, produced using the antisolvent method, have often been used for the wet encapsulation of hydrophobic bioactive ingredients (e.g., curcumin) through the production of Pickering emulsions [19,23]. Furthermore, kafirin has been successfully used as sole wall material for the dry encapsulation (e.g., dried particles) of catechin and sorghum tannins by freeze-drying [24], or in combination with sodium caseinate for the encapsulation of soybean oil by spray-drying [25]. Recently, we reported the development of fish oil-loaded electrosprayed particles using kafirin as the sole wall material [26]. Nonetheless, to the best of our knowledge, the ability of kafirin to protect fish oil encapsulated by electrospraying from lipid oxidation and the comparison with the use of zein have not yet been studied.

Therefore, this work aimed at investigating the oxidative stability of fish oil-loaded capsules produced by electrospraying using zein or kafirin as hydrophobic wall materials. First, the physicochemical properties of the obtained capsules (size, morphology, encapsulation efficiency, glass transition temperature) were evaluated. Secondly, electron paramagnetic resonance (EPR) and differential scanning calorimetry (DSC) were used to study the oxidative stability of the encapsulates.

## 2. Materials and Methods

### 2.1. Materials

Fish oil (FO) (Omega Oil 1812 TG Gold) was acquired from BASF Personal Care and Nutrition GmbH (Illertissen, Germany). Zein (Z) from maize, grade Z3625 (Lot# SLCD0046) with protein content 94.0 ± 0.4% (conversion factor nitrogen-to-protein of 6.25), was purchased from Sigma-Aldrich S.A. (Madrid, Spain). Spin trap (α-Phenyl-N-tertbutyl nitrone, PBN) was purchased from Sigma Aldrich (Madrid, Spain). The rest of the reagents used were of analytical grade.

### 2.2. Isolation of Kafirin

Kafirin (K) was isolated from decorticated grain from a commercial sorghum hybrid (F1000) (NuLife Market, Scott City, KS, USA) as described by Cetinkaya et al. [26]. The protein content of the isolated kafirin was determined via nitrogen combustion with a Leco FP-628 nitrogen determinator (Leco Corporation, St. Joseph, MI, USA) with a nitrogen-to-protein conversion factor of 6.25. Protein content was 89.2% (as-is basis).

### 2.3. Characterization of Kafirin

For RP-HPLC, isolated kafirin was dissolved in 60% t-butanol (*v*/*v*) with 0.5% sodium acetate (*w*/*v*) and 2% β-mercaptoethanol (*v*/*v*). After being dissolved, the sample was alkylated with 4-vinylpyridine and analyzed with a C18 column as described by Bean et al. [27]. For size exclusion analysis (SEC), the kafirin isolate was dissolved (2 mg/mL) in a 50 mM Tris-borate buffer, pH 7.0, containing 1% SDS (*w*/*v*), and analyzed as described by Ioerger et al. [28]. Molecular weight marker proteins bovine serum albumin (66 kDa), carbonic anhydrase (29 kDa), and lysozyme (14.3 kDa) were analyzed under the same conditions as the kafirin isolate. Results are shown in the Supplementary Materials.

### 2.4. Preparation of Electrospraying Solutions

Fish oil-in-water emulsions were produced to achieve an oil load of the dry capsules of ~13 wt.%. For this purpose, fish oil (2.0 wt.%) was dispersed in ethanol/water solvent (85/15, *v*/*v*) containing kafirin or zein (15 wt.%) using a hand disperser, POLYTRON^®^ PT1200E, (Kinematic Inc., New York, NY, USA), set at 18,000 rpm. The fish oil was added during the first minute of mixing, and the total mixing time was 2 min. The zein or kafirin was dissolved in the ethanol/water solvent (85/15, *v*/*v*) by magnetic stirring (300 rpm) for 1 h at room temperature. To investigate the oxidative stability of the encapsulated systems by EPR, PBN was added to fish oil as an ethanol solution (50 mg/mL) to obtain a final concentration of 30 mM of PBN in the lipid phase.

### 2.5. Production of Electrosprayed Capsules

Immediately after production, emulsions were monoaxially electrosprayed using the SpinBox^®^ electrospinning device (Bioinicia S.L., Valencia, Spain), which consists of a drying chamber equipped with a variable high-voltage power supply, a syringe pump, and a stainless-steel collector plate. The infusing flow rate was fixed to 0.6 mL/h regardless of the protein-based emulsion, the voltage applied varied between 15 and 18 kV, and the distance from the injector to the collector was kept at 15 cm. Electrospraying was carried out using a 16 G needle at ambient conditions (19–23 °C, 22–50% RH) in batches of 30 min. The nanocapsules were collected from the different batches and gently mixed to ensure that the analyzed samples were homogeneous and representative of the obtained material. Kafirin and zein nanocapsules without fish oil were designated as K-NFO and Z-NFO, respectively. Kafirin and zein nanocapsules loaded with fish oil were designated as K-FO and Z-FO, respectively. The capsules were stored in airtight flasks, at −80 °C in the dark until further analysis.

### 2.6. Characterization of Electrosprayed Capsules

#### 2.6.1. Scanning Electron Microscopy

A FESEM microscope (LEO 1500 GEMINI, Zeiss, Oberkochen, Germany) was used to determine the morphology of the capsules by scanning electron microscopy (SEM). The samples were placed on carbon tape and carbon coated as described in our previous work [29]. The SEM images were acquired in the range of 500×–15 k× magnification with a 5 kV accelerating voltage. The particle size distributions and mean diameters were determined by measuring 160 randomly selected capsules using the ImageJ software 1.47 (National Institute of Health, Bethesda, MD, USA). The polydispersity index (*PdI*) was calculated as *PdI* = (σ/D)^2^, where σ corresponds to the standard deviation of the diameter, and D is the mean diameter of the capsules.

#### 2.6.2. Encapsulation Efficiency (EE)

The encapsulation efficiency of the electrosprayed capsules was measured as described in our previous work, with some modifications, including a washing method and the removal of non-encapsulated fish oil from the surface of the capsules [16]. In brief, ca. 25 mg of nanocapsules were placed inside a funnel made from grade 1 filter paper with 11 µm particle retention (Whatman, Buckinghamshire, UK), and then 5 mL of isooctane was gently poured over the capsules. The absorbance of the filtrate solvent was measured in triplicate at 284 nm using a NanoDropTM One/OneC Microvolume UV–Vis spectrophotometer (Thermo Fisher Scientific, Waltham, MA, USA). The amount of extractable oil contained in the filtrate was determined from a calibration curve (R^2^ = 0.99) prepared by dissolving various quantities of fish oil in isooctane (0.005–0.15 mg/mL). The *EE* was calculated as follows:

(1) $$EE\;\%=\frac{A-B}{A}\cdot 100$$

where *A* refers to the theoretical oil load of the nanocapsules (g), and *B* to the extractable oil (g). The measurements were carried out in triplicate.

#### 2.6.3. Attenuated Total Reflection–Fourier Transform Infrared (ATR–FTIR) Spectroscopy

FTIR analysis was performed using a Nicolet iS50 FT-IR (Thermo Fisher Scientific, Waltham, MA, USA). To conduct the measurement, enough of each sample was placed on the diamond ATR to ensure proper coverage. The spectra were recorded over a wavenumber range of 4000–400 cm^−1^ with 32 scans at a resolution of 4 cm^−1^, at room temperature (20 ± 2 °C).

#### 2.6.4. Raman Spectroscopy

The Raman spectra of electrosprayed nanocapsules were acquired using a DXR3 Raman microscope (Thermo Fisher Scientific, Waltham, MA, USA) equipped with Omnic 9.12.928 software. The spectra were collected with a laser wavelength of 532 nm, a preview exposure time of 5 s, a number of exposures of 20 s, a laser power of 8 mW, 10× magnification, a pinhole size of 25, and an aperture within the range of 4000 to 400 cm^−1^.

#### 2.6.5. Differential Scanning Calorimetry (DSC)

The glass transition temperature (T_g_), which is a crucial property for electrosprayed nanocapsules, was determined using the modulated differential scanning calorimetry (MDSC) method using a Discovery DSC 250 (TA Instruments Ltd., New Castle, DE, USA) following the protocol described by García-Moreno et al. [30]. For obtaining the sample thermograms, 3 ± 0.15 mg of capsules were hermetically sealed in an aluminium pan (Tzero aluminium hermetic pans, TA Instruments, New Castle, DE, USA), while an empty hermetically sealed aluminium pan was used as a reference. Samples were initially cooled down and equilibrated for 10 min at −80 °C, and then heated to 200 °C with a heating ramp of 10 °C/min under a nitrogen gas at 50 mL/min flow rate. The glass transition midpoint of the samples was detected at the step change point using TRIOS software (V5.6.87) (TA Instruments, New Castle, DE, USA). The measurements for each sample were conducted in duplicate.

### 2.7. Oxidative Stability of Electrosprayed Capsules

#### 2.7.1. Electron Paramagnetic Resonance (EPR)

The EPR spectra of the encapsulated systems containing the spin trap (PBN) were recorded using a MiniScope MS5000 (Bruker, Rheinstetten, Germany) at ambient temperature. The modulation amplitude used in all EPR measurements was kept constant in each determination at 0.2 mT. Quartz EPR tubes (5 mm OD) were filled with powder to obtain a sample height of ~3.5 cm to ensure that the part of the tube inside the resonant cavity of the spectrometer was completely filled with sample [31]. Thus, the volume of the capsules was kept constant for the analyses. Three tubes were prepared per sample and subsequently stored at 25 °C in the dark for 25 days. Additionally, the spin probe PBN was added to pure fish oil to obtain a concentration of 30 mM in the oil. The EPR spectra of fish oil containing PBN were measured by soaking a filter paper in the oil and placing it in an EPR tube.

#### 2.7.2. Differential Scanning Calorimetry (DSC)

For the assessment of the oxidative stability of the nanocapsules, the differential scanning calorimetry (DSC) technique, using DSC 250 (TA Instruments Ltd., New Castle, DE, USA), following a previously described oxidation induction time (OIT) protocol [32], was used. The DSC apparatus was calibrated using high-purity indium, and measurements were conducted using TA Instrument Trios software (V5.6.0.87) (TA Instruments, New Castle, DE, USA). For each measurement, 3 mg of sample was weighed into an open aluminium pan and placed in the DSC tray, with an empty aluminium pan with an open lid serving as the reference.

In the initial phase, the experiments were conducted under a nitrogen atmosphere with a 50 mL/min flow rate. The samples were equilibrated at 10 °C for 5 min, and then heated to 140 °C at a rate of 5 °C/min, followed by a 5 min isothermal hold. Subsequently, the purge gas was switched to oxygen (99.995% purity) at a 50 mL/min flow rate. During this phase, the DSC cells were maintained isothermally for 120 min while data were recorded.

The oxidation of the capsules was quantified by measuring the area under the isothermal DSC curves [32,33,34]. A calibration curve for non-encapsulated fish oil was also created for comparison with the encapsulated fish oil.

### 2.8. Statistical Analysis

Data were subjected to analysis of variance (ANOVA) using OriginPro 2021 (version 9.8.0.200, OriginLab Corporation, Northampton, MA, USA). Tukey’s multiple range test was used to determine significant differences between mean values. Differences between mean values were considered significant at a level of confidence of 95% (*p* < 0.05) and indicated with different lowercase letters.

## 3. Results and Discussion

### 3.1. Characterization of the Electrosprayed Capsules

#### 3.1.1. Morphology, Particle Size Distribution, and Encapsulation Efficiency

**Figure 1:**
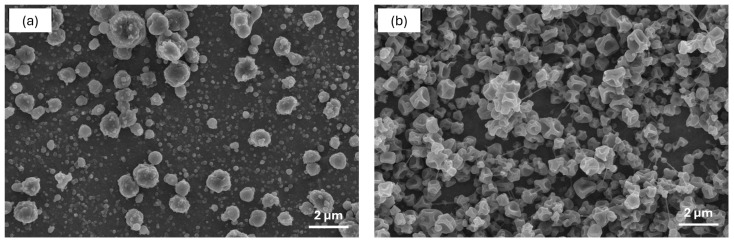
SEM images of fish oil-loaded capsules (13 wt% fish oil) produced by monoaxial electrospraying. K-FO sample (**a**); Z-FO sample (**b**).

As shown in Figure 1, monoaxial electrospraying processing resulted in a discrete distribution of spherical particles when kafirin was used as the encapsulating agent (Figure 1a), which is in line with the results previously reported by Cetinkaya et al. [26], whilst zein-based nanocapsules showed a dented surface (Figure 1b), as observed in other studies where the electrospray of zein solutions in ethanol was conducted [35]. These results contrast with the morphology observed in previous studies for omega-3-loaded nanocapsules produced by electrospraying with zein as the encapsulating agent, where mostly spherical particles were produced [15,16]. However, it must be considered that the aforementioned authors produced the nanocapsules using pilot-plant electrospraying assisted by pressurized gas (EAPG) technology, where the infeed emulsion is first mechanically atomized within the high-voltage electrostatic field, contrary to the electrohydrodynamic atomization achieved at lab-scale electrospraying processing reported in the current study, which might explain our results.

**Figure 2:**
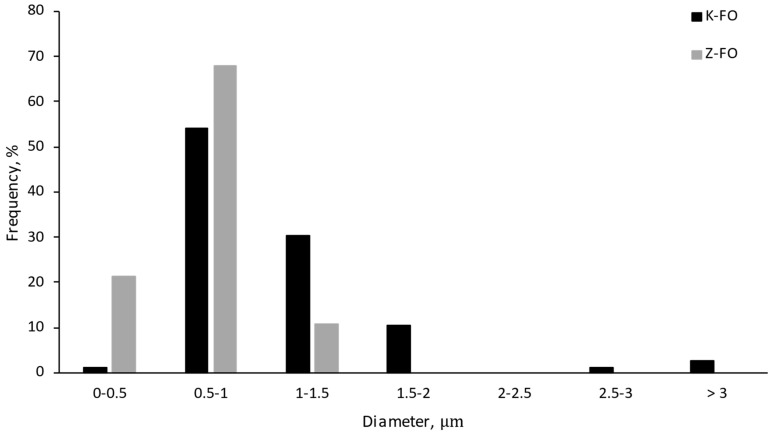
Particle size distribution of fish oil-loaded capsules produced by monoaxial electrospraying: K-FO (black) and Z-FO (grey).

As for the particle size distribution of the fish oil-loaded nanocapsules, kafirin resulted in significantly larger capsules (mean diameters of 1.1 ± 0.5 µm and 0.7 ± 0.2 µm for K-FO and Z-FO, respectively) with a broader particle size distribution (*PdI* of 0.21 for K-FO vs. 0.08 for Z-FO) (Figure 2) despite the protein content of the infeed emulsions (15 wt.%), and the infeed flow rates (0.6 mL/h) were the same, irrespective of the hydrophobic protein used as the encapsulating agent (i.e., kafirin or zein). In addition, although it is known that higher voltages applied during electrospraying processing result in smaller capsules due to increased electrostatic charge repulsion [5], the variation in the voltage applied during processing (15–18 kV) was not different enough to have such a significant impact on the particle size of the nanocapsules. Therefore, we hypothesize that the differences observed could be attributed to the different hydrophobicity of the native proteins. Although both kafirin and zein, as hydrophobic proteins, are soluble in alcohol-based solutions, kafirin is relatively more hydrophobic compared to zein [23,36], thus requiring larger amounts of ethanol to result in a homogeneous solution [37]. Ethanol plays an important role by improving the incorporation of poorly water-soluble proteins into oil-in-aqueous ethanol emulsion [38]. Kafirin solubility in different concentrations (10 to 100%) at 2 mg/mL revealed that the maximum solubility occurs at 70% ethanol. This behaviour is related to the amphiphilic nature of kafirin in a way that the hydrophilic segment of the protein was oriented outward at 70% ethanol [39], while at 85% ethanol, the solubility decreases to about 60%. Another study showed an increase in the solubility of (4 wt.%) zein in ethanol content up to 90% (solubility of zein at 85% ethanol is 70%). The high content of non-polar amino acid residues in zein structure leads to this solubility behaviour [40,41] Thus, kafirin might be dispersed but not totally solubilized in the solvent used in this study, thus explaining the larger protein aggregates observed.

The encapsulation efficiency of Z-FO electrosprayed nanocapsules was 92.4 ± 3.8%, while the EE obtained for K-FO nanocapsules was 38.4 ± 6.4%. This indicates that fish oil was encapsulated 2.4 times more in the zein matrix than in kafirin-based nanocapsules. Nevertheless, it should be mentioned that the EE value obtained for the fish oil-loaded kafirin capsules was considerably lower when compared to our previous work, where EE of 94.0 ± 2.5% was reported [26]. This difference might be attributed to the lower kafirin content used in our previous study (10 wt.%), which might facilitate the dispersion of fish oil in the continuous phase during homogenization, leading to better oil entrapment. In any case, it should also be pointed out that kafirin might also be removed from the surface in the current study when evaluating EE by washing with isooctane, which increases UV-absorbance, underestimating the EE value. This latter statement agrees with the results from ATR-FTIR spectra, as discussed below.

#### 3.1.2. ATR-FTIR

The FTIR spectra of electrosprayed fish oil-loaded nanocapsules were compared with the free fish oil and control capsules without fish oil to identify and detect any potential interaction between materials. The fatty acid composition of fish oils specifies the band position and shape of the FTIR spectrum. Consequently, any changes in the fatty acids proportion in the triglyceride molecules result in band shifts [42,43].

**Figure 3:**
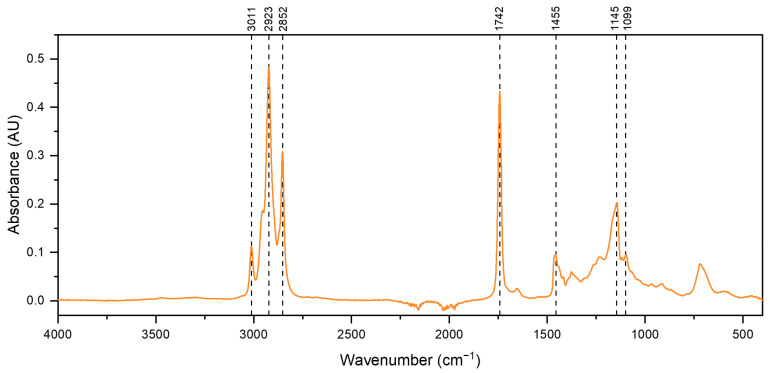
ATR-FTIR spectrum of fish oil (FO).

The FTIR spectrum of fish oil (Figure 3) exhibits a triplet band at 2800–3000 cm^−1^, which is attributed to the C–H stretching modes of the methyl and methylene backbone of lipids [44,45]. A band at 3011 cm^−1^ corresponds to the C–H stretching of cis–alkene–HC=CH– from unsaturated fatty acids. The sharp band at 2923 cm^−1^ is attributed to methylene (CH_2_) groups, and the band at 2852 cm^−1^ represents methyl (CH_3_) (both symmetrical stretching). Another sharp band at 1742 cm^−1^ is assigned to the C=O stretch of the ester functional group from ethyl esters of lipids and fatty acids. The band at 1455 cm^−1^ is assigned to the asymmetrical deformation scissor from methylene (CH_2_). The band at 1145 cm^−1^ is attributed to CH_2_ out-of-plane deformation modes, and a small band at 1099 cm^−1^ is assigned to C–O–C symmetrical stretches [44,46,47].

**Figure 4:**
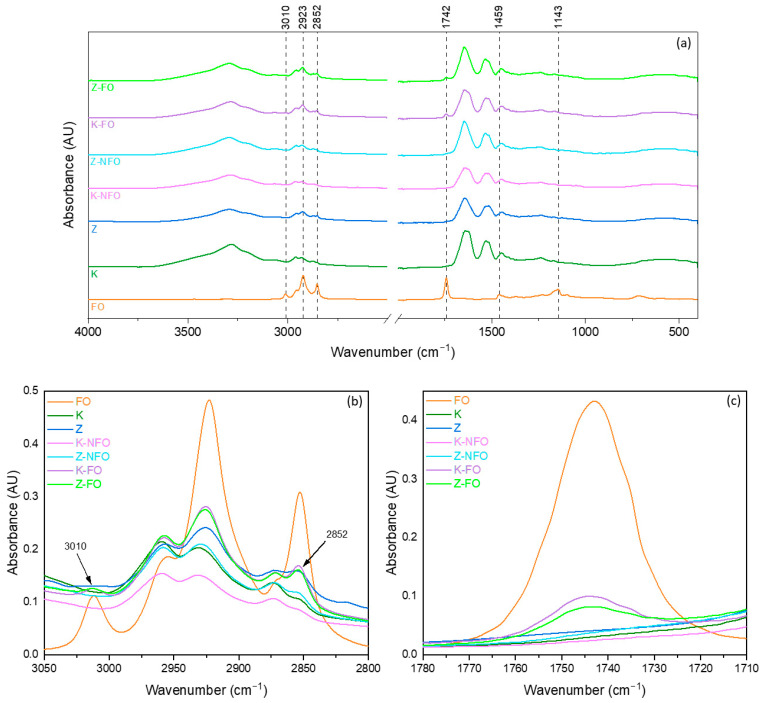
FTIR spectra of fish oil (FO), kafirin (K), zein (Z), electrosprayed zein capsules (Z-FO), electrosprayed kafirin capsules without fish oil (K-NFO) or loaded with fish oil (K-FO), and electrosprayed zein capsules without fish oil (Z-NFO) or loaded with fish oil (Z-FO) (**a**); spectra expanded region from 3050 to 2800 cm^−1^ (**b**); and spectra expanded region from 1780 to 1710 cm^−1^ (**c**).

In the FTIR spectrum of kafirin (K) (Figure 4), a broad band was observed at 3280 cm^−1^ and in the FTIR of zein at 3292 cm^−1^, which is the characteristic band of the protein assigned to amid I stretching (N–H stretching). The same broad band was also observed in the control and fish oil-loaded nanocapsules. The FTIR spectrum of kafirin displays bands at 2959 and 2931 cm^−1^, and in zein (Z), it appears at 2957 and 2924 cm^−1^, related to the asymmetric stretching vibration of =C–H and NH_3_. Two major bands at the 1645 and 1532 cm^−1^ could be seen in both spectra of kafirin and zein, which are typical from proteins amide I and amide II functional groups. The same bands were also observed in the spectra of electrosprayed nanocapsules. A small band at 1447 and 1239 cm^−1^ is assigned to CH_2_ bending vibration, and the band at 1239 cm^−1^ is the amide III together with C–N stretching of peptide bonds [25,48,49]. Shifts in the band position in K-NFO from 2931 to 2925 cm^−1^ in the K-FO sample were observed, as well as in Z-NFO from 2929 to 2925 cm^−1^ (Figure 4b). The mentioned shifts in the characteristic band of the N–H stretching to lower wavenumbers in the fish-loaded nanocapsules suggested the formation of hydrogen bonds, as seen in the study in [49], where kafirin and polylactic acid were electrospun to encapsulate clove essential oil (CEO). The spectra of K-FO show a band at 1743 cm^−1^, which is characteristic of lipids and fatty acids (fish oil) and is assigned to the C=O stretched of the ester functional group. This band is more pronounced in the K-FO than Z-FO nanocapsules, confirming the higher encapsulation efficiency of fish oil measured for Z-FO, comparatively to K-FO nanocapsules (Figure 4c). Nevertheless, the small difference observed in the band at 1743 cm^−1^ between K-FO and Z-FO does not correlate with the considerable difference obtained in the EE values for these capsules. This fact indicates that although the fish oil was better entrapped in zein-based capsules, when compared to kafirin-based capsules, the markedly low EE value obtained for kafirin-based capsules might be underestimated due to the potential removal of kafirin when washing with isooctane.

#### 3.1.3. Raman Spectroscopy

**Figure 5:**
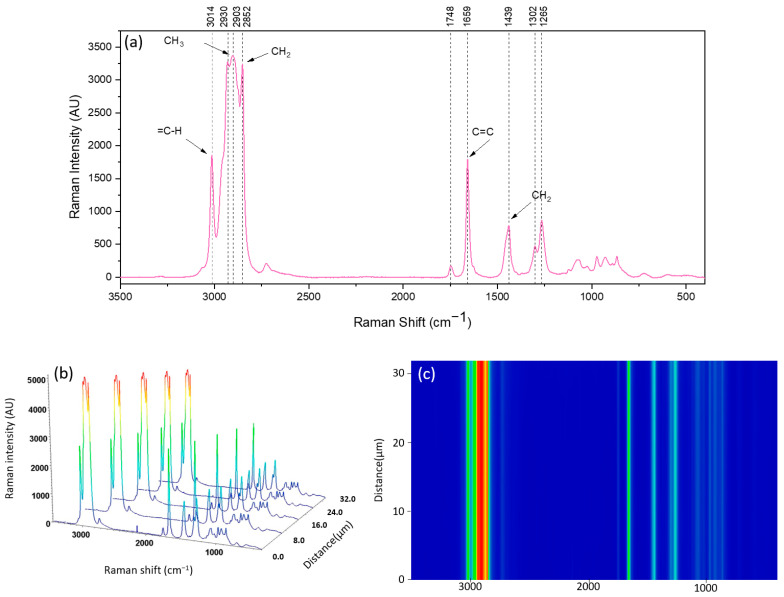
Raman spectra of fish oil (FO) (**a**); 3D image spectra from line-scanning Raman mapping across the surface of FO (**b**); and corresponding contour map image (**c**).

The Raman spectrum of free fish oil (FO) and a 3D image of line mapping across about 30µm of FO and its counter map image are shown in Figure 5. In the region ranging between 3000 and 2800 cm^−1^, several bands can be seen associated with the C–H stretching vibrations. The peak observed at 3014 cm^−1^ corresponds to the stretching of =C–H (of cis–HC=C–H) groups [46]. The band at 2930–2903 cm^−1^ is associated with CH_3_ groups, while the peak at 2852 cm^−1^ is assigned to CH_2_ groups [50]. The peak at 1748 cm^−1^ contributes to the C=O stretch, the peak at 1659 cm^−1^ is related to the stretching vibration of the C=C stretch, and the peak around 1439 cm^−1^ signifies the CH_2_ scissoring groups. Furthermore, the peaks at 1302 cm^−1^ and 1265 cm^−1^ are assigned to CH_2_ bending and symmetric =C–H rock (=C–H deformation), respectively [32,51,52]. As expected, the spectra of fish oil across the line mapping show the same peaks with the same intensity (Figure 5b,c).

**Figure 6:**
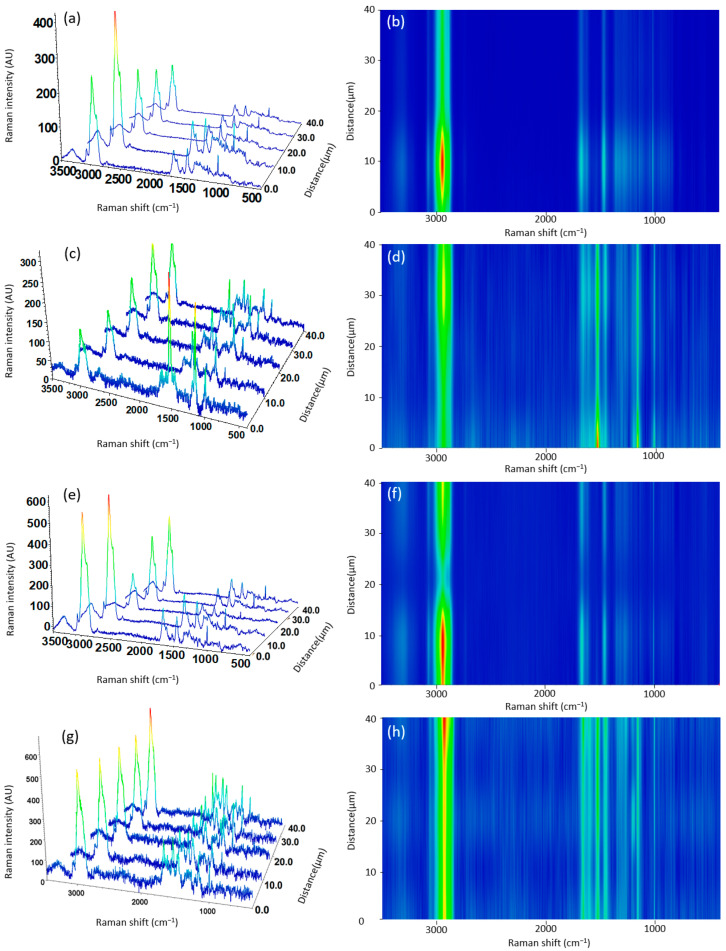
Three-dimensional image spectra from line-scanning Raman mapping across the surface of the electrosprayed (**a**) kafirin control (K-NFO), (**c**) zein control (Z-NFO), (**e**) fish oil-loaded kafirin (K-FO), (**g**) fish oil-loaded zein capsules (Z-FO); and (**b**,**d**,**f**,**h**) corresponding contour map images.

In Figure 6, the Raman spectra of both electrosprayed kafirin (Figure 6a) and zein (Figure 6c) nanocapsules show typical peaks for Raman spectra of proteins [53]. For instance, a main intense peak can be seen around 2934–2929 cm^−1^, assigned to N–H stretching from amide groups [53]. In the region of 1656 cm^−1^, the peak is assigned to C=O Amide I, the peak at 1450 cm^−1^ is assigned to C-H deformation, and the peak at 10 cm^−1^ is attributed to aromatic residues of Phenylalanine [53,54]. The Raman spectra of electrosprayed kafirin (Figure 6e), and zein (Figure 6g) nanocapsules loaded with fish oil showed no noticeable or major shifts compared to kafirin and zein capsules without fish oil, therefore suggesting the successful encapsulation of fish oil. However, it is to be noted that the intensity of some of the Raman peaks is higher in the capsules loaded with fish oil, particularly for the Z-FO, as evidenced in contour map images (Figure 6h), probably due to the higher encapsulation of fish oil in these nanocapsules.

#### 3.1.4. Glass Transition Temperature (T_g_)

**Figure 7:**
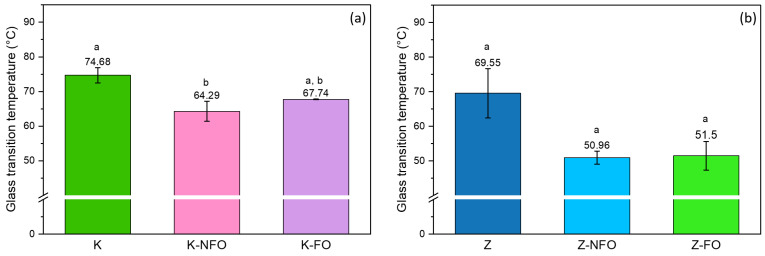
Glass transition temperature of (**a**) kafirin samples and (**b**) zein samples. Different superscript letters indicate significant differences (*p* < 0.05) among the samples.

Figure 7 shows the T_g_ of the proteins and the protein electrosprayed nanocapsules containing fish oil. Both protein-based capsules are in a glassy state at room temperature, which is preferred as it restricts the diffusion of oxygen and other prooxidants and enhances the oxidative stability of the fish oil. The zein protein had a glass transition of about 70 °C, whereas the T_g_ of the zein capsules was reduced to about 20 °C. The T_g_ of the kafirin protein is close to zein (75 °C), whereas the reduction in the T_g_ was lower for the kafirin capsules (about 10 °C in comparison to the protein). The T_g_ values of the proteins are similar to those previously reported in the literature. The glass transition temperature of kafirin was reported in the literature to range from 40 °C to 233.8 °C, and typically higher than the T_g_ of zein [36,55]. The broad range of kafirin’s T_g_ is probably due to the moisture content of kafirin, which plasticizes the prolamins and other proteins and substantially reduces their T_g_, as well as due to the opposing interpretations of the endotherm that denotes its glass transition [36]. It should be noted that the addition of fish oil did not affect the T_g_ of the nanocapsules (Figure 7).

### 3.2. Oxidative Stability of Electrosprayed Capsules

#### 3.2.1. Electron Paramagnetic Resonance (EPR)

**Figure 8:**
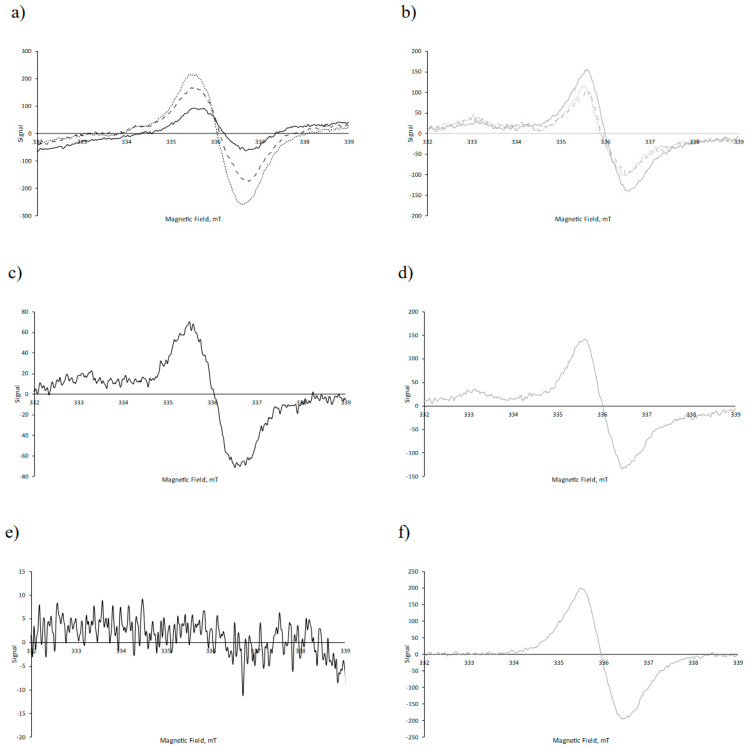
EPR spectra of protein samples at day 0: (**a**) kafirin capsules loaded with fish oil (K-FO), (**b**) zein capsules loaded with fish oil (Z-FO), (**c**) kafirin capsules without fish oil (K-NFO), (**d**) zein capsules without fish oil (Z-NFO), (**e**) kafirin protein powder (Z), and (**f**) zein protein powder (Z). In (**a**,**b**), day 0 (solid line), day15 (dashed line), and day 25 (dotted line).

An evaluation of the protective effects of the nanoencapsulation for lipid oxidation was attempted through the detection of lipid radicals by EPR spectroscopy combined with spin trapping. Radicals are central-chain-carrying intermediates in the autoxidation of polyunsaturated fatty acids. Radicals formed during lipid oxidation have a short lifetimes, leading to low steady-state concentrations, which prevent the direct detection of these radicals by EPR in most food systems [56]. The EPR spin-trapping technique, which is based on spin traps that react with lipid radicals, allows for the indirect monitoring of lipid oxidation through the generation and detection of long-lived radical adducts [57]. EPR spin trapping has been used to evaluate the oxidative stability of bulk oils [58], oil-in-water emulsions [59,60,61,62], and fish oil-loaded nano-microcapsules produced either by electrospraying or spray-drying [29,31,63]. N-tert-butyl-a-phenyl nitrone (PBN), a nitrone spin trap, was dissolved in the fish oil. The EPR spectrum of PBN lipid radical adducts formed in encapsulated fish oil has previously been reported to consist of three broad lines with the typical coupling for nitroxyl radicals due to the nitrogen nucleus [29,58,62,63]. However, the EPR spectra obtained in this study for the fish oil-loaded electrosprayed capsules with zein or kafirin as wall materials, using PBN as spin-trap, did not show the typical three broad lines (Figure 8). The absence of the EPR spectrum of spin-trapped lipid radicals do not, however, rule out that lipid oxidation did not occur in the encapsulated fish oils, since EPR-detectable spin adducts have previously been found to be unstable in the presence of high levels of radicals that may be present in fish oils that undergo extensive oxidation. This could have been the situation during the preparations of the fish oil emulsions or during the subsequent electrospraying.

Despite the absence of evidence of spin-trapped lipid radicals, the EPR spectra of fish oil-loaded capsules (K-FO, Z-FO) consisted of broad peaks, which are practically the same as EPR spectra of electrosprayed zein or kafirin proteins without fish oil (K-NFO, Z-NFO) (Figure 8a–d). These spectra are characteristic of large and partially immobilized protein spin adducts or radicals trapped in glassy encapsulation materials [64]. Indeed, it was observed that electrospraying processing induced the formation of kafirin protein radicals, which were not observed in the native kafirin (Figure 8c,e), and that the concentration of the protein radicals in the commercial zein increased due to electrospraying as indicated by the increase in the intensity (Figure 8d,f). The intensity of the broad peaks increased during 25 days of storage for fish oil-loaded kafirin capsules, whereas a slight decrease was observed for zein capsules loaded with fish oil (Figure 8a,b). In line with these results, Amft et al. [65] obtained an EPR spectrum of commercial zein containing a single broad peak, which was likely due to the superposition of several carbon- and nitrogen-centred radical species from zein proteins.

Altogether, these results indicate that radicals are generated during the electrospraying process, where the lipid-derived radicals are eventually trapped and immobilized in the glassy encapsulation matrix. The further increase in the intensity of these immobilized radicals in the kafirin capsules indicates that oxidation can progress in this system.

#### 3.2.2. Differential Scanning Calorimetry (DSC)

**Figure 9:**
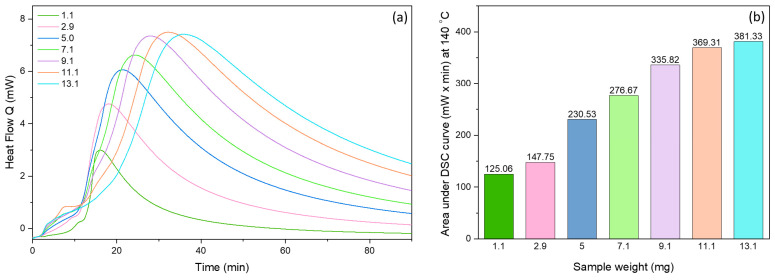
(**a**) Isothermal DSC oxidation curves of non-encapsulated fish oil at different oil magnitudes at 140 °C and oxygen flow of 50 mL/min. (**b**) Area under the isothermal oxidation curve for non-encapsulated fish oil at different oil concentrations.

Figure 9 shows the DSC oxidation curves for the non-encapsulated fish oil at different magnitudes (1–13 ± 0.1 mg) at an isothermal temperature of 140 °C. A large exothermic peak is clearly shown, which considerably increases as the quantity of the sample increases. The area under the isothermal curve can be used to quantify and compare the oxidative stability of the oil [32,33,34]. The area under the isothermal curve for 13.1 mg oil was nearly three-fold higher (381.33 mW × min) in comparison to the area of the 1.1 mg oil (125.06 mW × min).

**Figure 10:**
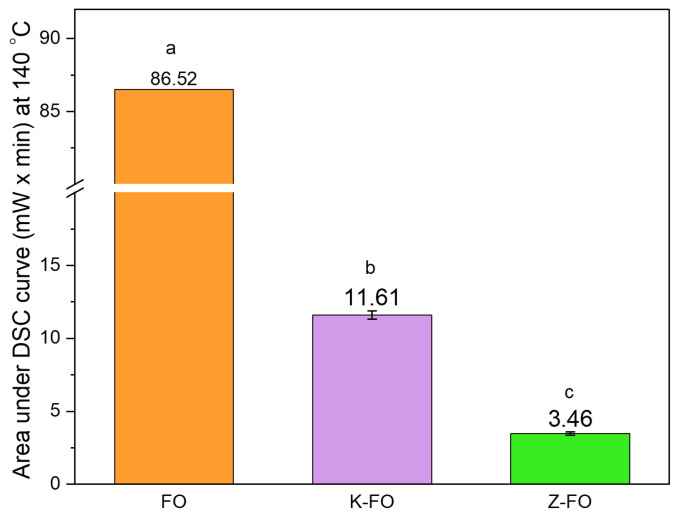
Isothermal DSC results at 140 °C and oxygen flow of 50 mL/min. Area under the isothermal oxidation curve for non-encapsulated FO, fish oil-loaded kafirin, and fish oil-loaded zein nanocapsules. Different superscript letters indicate significant differences (*p* < 0.05) among the samples.

The oxidative stability of encapsulated fish oil was compared with non-encapsulated fish oil using the prepared calibration curve. The encapsulation of fish oil by electrospraying using kafirin or zein as wall materials protected it from oxidation (Figure 10). The area under the isothermal curve for non-encapsulated oil was nearly 7.45 times higher and 25 times higher, in comparison to the area of the encapsulated oil within kafirin and zein nanocapsules, respectively. In other words, zein nanocapsules showed 3.3 times more protection against oil oxidation than kafirin nanocapsules at 140 °C and under oxygen flow. This reveals the high oxidative stability of encapsulated fish oil that is provided by the protein matrix by enhancing the impermeability to oxygen and by providing thermal stability.

The difference between the oxidative stability of K-FO and Z-FO nanocapsules might be due to the different lower encapsulation efficiency of the oil within kafirin capsules, and probably the slightly higher surface oil of the kafirin capsules as also observed by FTIR. Furthermore, previous studies showed that typically films produced from commercial zein had slightly better oxygen barrier properties than kafirin over a wide range of plasticizer concentrations (20%–40%) [66]. This was attributed to the free volume of their films as it can increase the oxygen permeability rate by up to six orders of magnitude. The much greater disulfide bond cross-linking in kafirin films resulted in areas of a larger intermolecular free volume than in commercial zein films [36,55,67]. Indeed, the average free volume substantially affects the oxygen diffusivity in glassy matrices [68]. Boerekamp et al. also showed that glucose syrup capsules present higher oxidative stability than dextran capsules, and this was attributed to the lower molecular weight of glucose syrup, which led to a lower free volume in the glassy matrix, reducing oxygen diffusivity [63]. Overall, the thermal and oxygen stability of the encapsulated fish oil indicates effective encapsulation with both protein matrices against degradation and oxidation.

## 4. Conclusions

The encapsulation of fish oil by monoaxial electrospraying was effectively carried out when using kafirin or zein as hydrophobic wall materials, resulting in mostly sub-micron capsules (>50% capsules for kafirin and >80% capsules for zein were below 1 µm). Moreover, EPR results indicate that oxidation occurred during the electrospraying process and the storage of the kafirin electrosprayed capsules loaded with fish oil. Nonetheless, spin trapping did not allow us to detect the presence of lipid radicals. The isothermal DSC results reveal that both kafirin- and zein-based electrosprayed capsules presented higher oxidative stability than non-encapsulated oil. Kafirin-based capsules were less oxidatively stable than zein-based capsules, which was attributed to the higher oil encapsulation efficiency obtained for zein capsules and the lower oxygen impermeability of kafirin when compared to zein. Overall, this study suggests that although kafirin might be considered an alternative hydrophobic encapsulating agent, it resulted in fish oil-loaded electrosprayed nanocapsules with lower oxidative stability than zein.

## Data Availability

The original contributions presented in the study are included in the article; further inquiries can be directed to the corresponding author.

## Supplementary Materials

The following supporting information can be downloaded at https://www.mdpi.com/article/10.3390/antiox13091145/s1.
