# Supplementary material for: Oxidative Stability of Fish Oil-Loaded Nanocapsules Produced by Electrospraying Using Kafirin or Zein Proteins as Wall Materials
Source: Antioxidants (Basel). 2024 Sep 23;13(9):1145. doi: 10.3390/antiox13091145 (PMC11428463; doi:10.3390/antiox13091145)
Supplement: Supplementary file 1 [file antioxidants-13-01145-s001.zip › antioxidants-3159943-supplementary.pdf]

SUPPLEMENTARY MATERIAL

S1. Characterization of the kafirin isolate

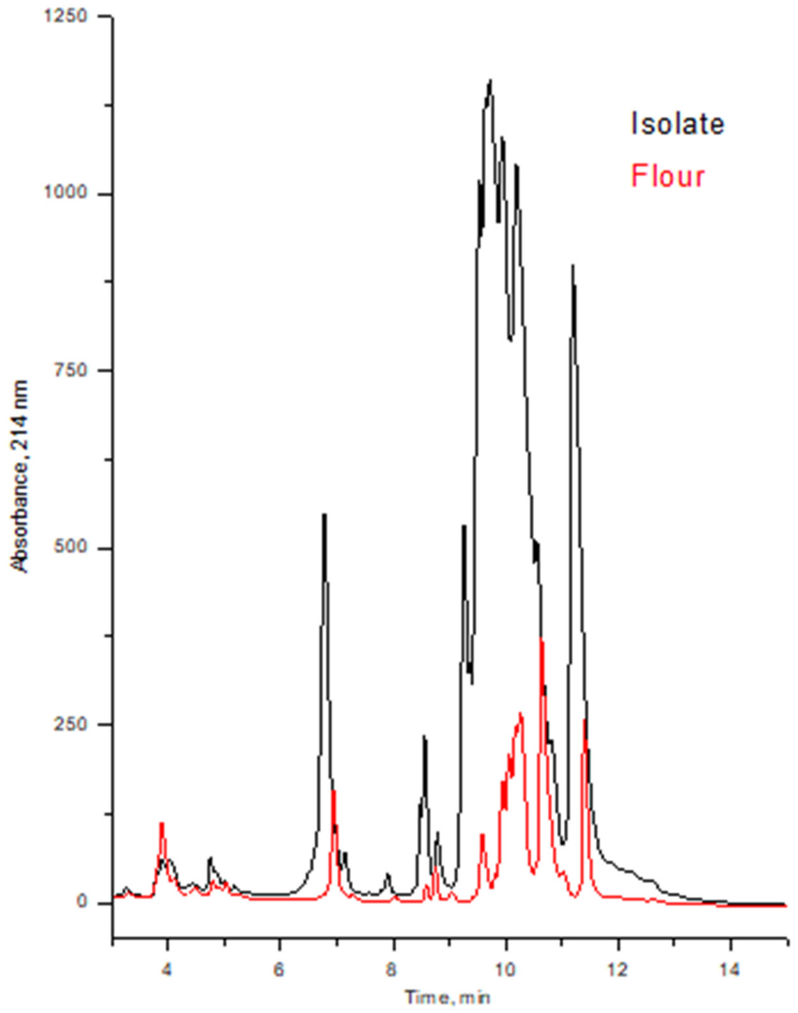

Fig. S1.1. RP-HPLC of decorticated flour and kafirin isolate (100 mg flour, 20 mg isolate)

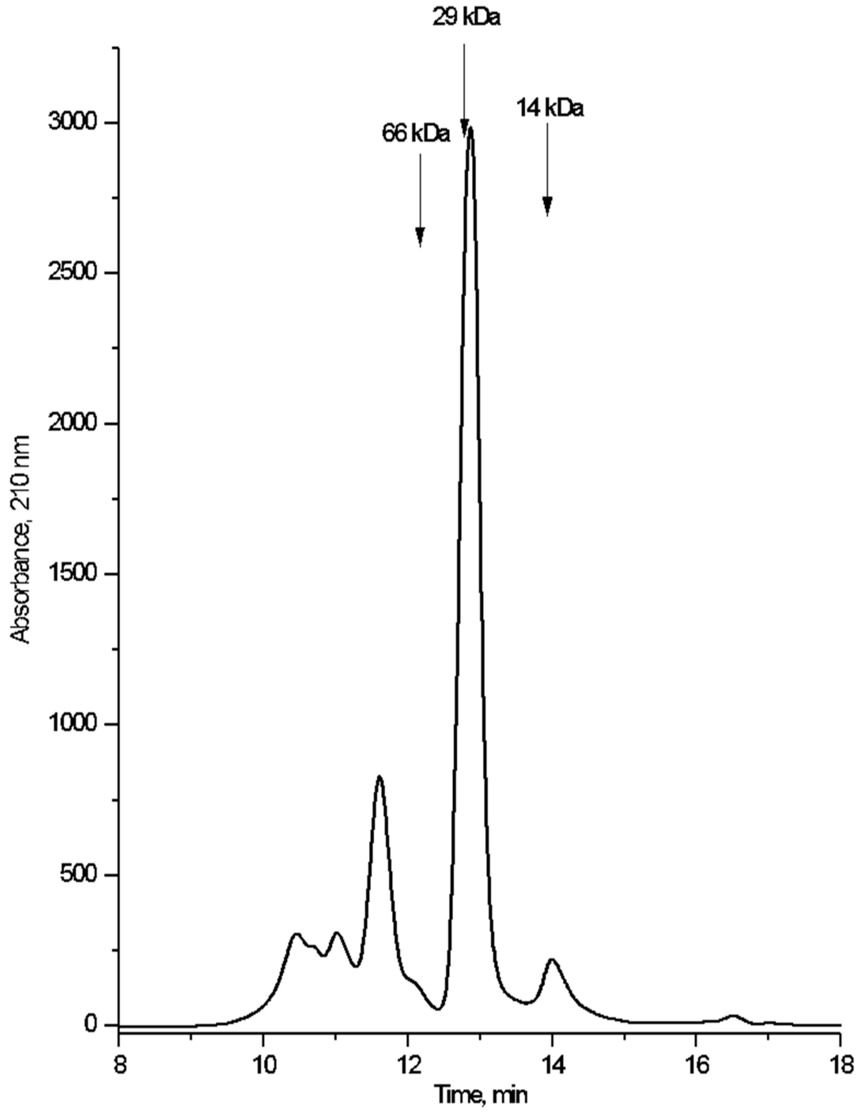

Fig. S1.2. Size exclusion chromatogram of kafirin isolate (2 mg/mL)

S2. Determination of glass transition temperature

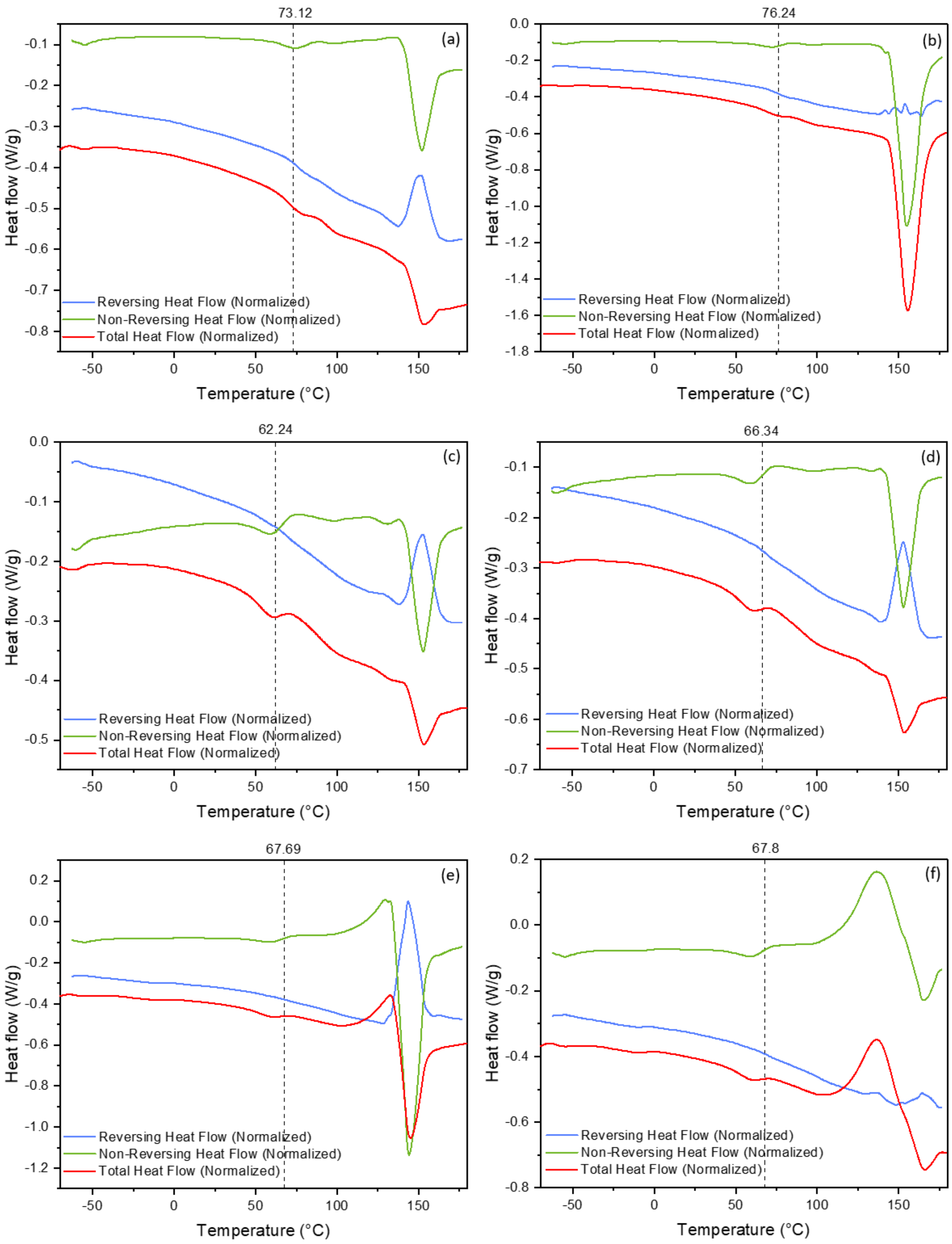

Figure S2.1. Modulated DSC, glass transition of kafirin powder (K) (a and b), kafirin electrosprayed without fish oil (K-NFO) (b and c), Kafirin electrosprayed containing fish oil (K-FO) (e and f).

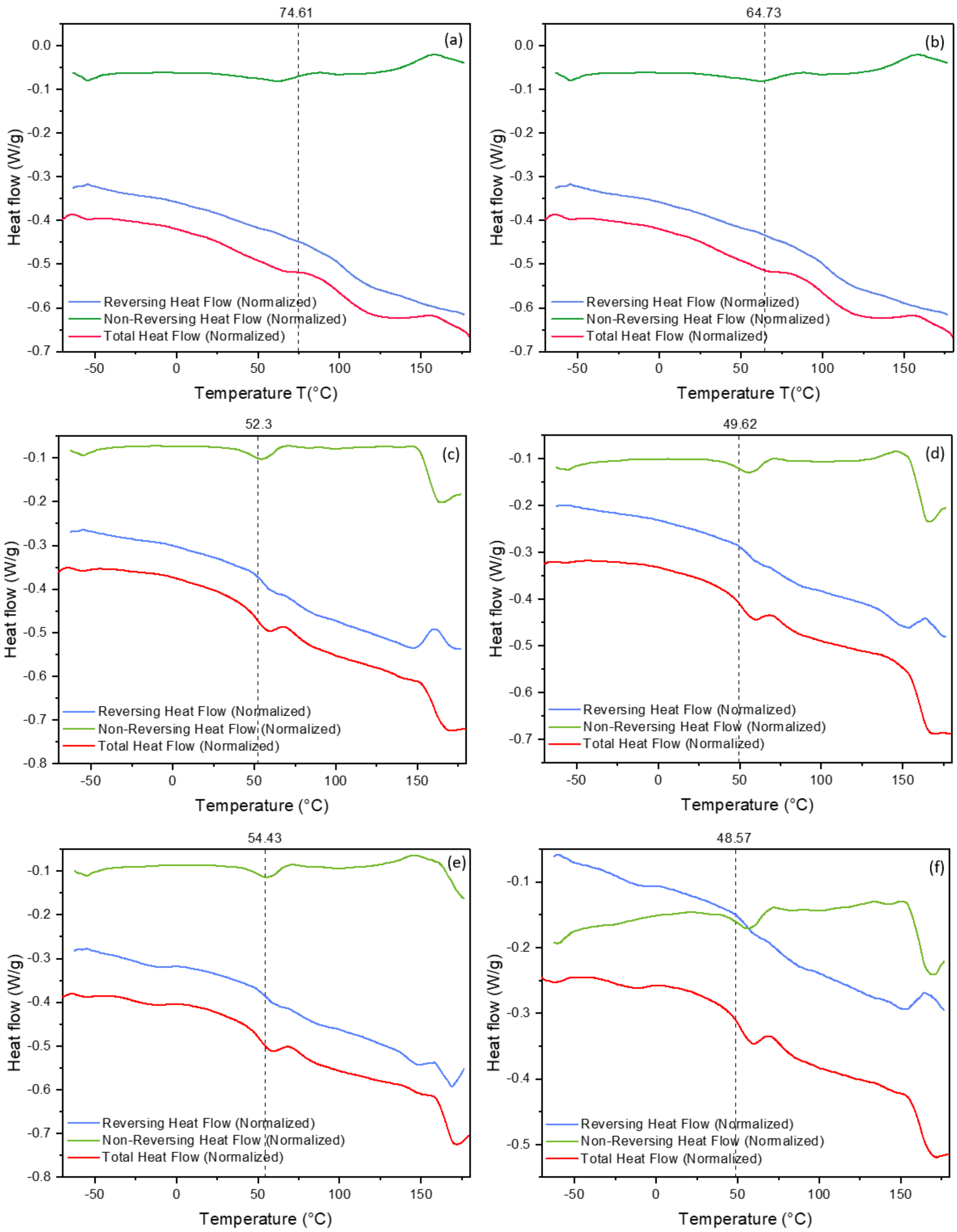

Figure S2. 2. Modulated DSC, glass transition of zein powder (Z) (a and b), zein electrosprayed without fish oil (Z-NFO) (c and d), zein electrosprayed containing fish oil (Z-FO) (e and f).
